# Carrier lifetime modulation on current capability of SiC PiN diodes in a pulsed system

**DOI:** 10.1186/s11671-023-03905-6

**Published:** 2023-10-16

**Authors:** Xingliang Xu, Lin Zhang, lianghui Li, Zhiqiang Li, Juntao Li, Jian Zhang, Peng Dong

**Affiliations:** 1 Microsystem and Terahertz Research Center, China Academy of Engineering Physics, Chengdu, China; 2 Institute of Electronic Engineering, China Academy of Engineering Physics, Mianyang, China; 3 School of Electronic Science and Engineering, University of Electronic Science and Technology of China, Chengdu, China

**Keywords:** Silicon carbide, PiN diodes, Carrier lifetime modulation, Pulse current, Defect engineering

## Abstract

Silicon carbide (SiC) PiN diode has shown substantial promise as the freewheel diode for switch protection in a pulsed system. In this paper, we investigate the carrier lifetime (*τ*) modulation on pulsed current capability of SiC PiN diodes. The carrier lifetime in 4H–SiC is modulated by the generation of the *Z*_1/2_ center through neutron irradiation. Surprisingly, we found that the pulsed current of SiC PiN diodes shows a limited improvement when the carrier lifetime (*τ*) increases from 0.22 to 1.3 μs, while is significantly promoted as the carrier lifetime increases from 0.03 to 0.22 μs. This changing trend is obviously different from the on-state resistance, which decreases with the increased carrier lifetime. The simulation result indicates that the heat generation (i.e., maximum temperature rise) inside the PiN diodes, especially in the drift layer, is remarkably aggravated in the pulse tests for *τ* < 0.1 μs, but which is significantly suppressed as carrier lifetime rises to 0.2 μs and above. Therefore, the dependence of pulsed current on carrier lifetime is ascribed to the heat generation resulting from the carrier lifetime controlled conductivity modulation effect, which hence affects the temperature rise and brings about the failure of SiC PiN diodes under high pulsed current.

## Introduction

Due to the excellent physical properties, silicon carbide (SiC) has aroused great attention all over the world both in academic and industrial fields. Its higher critical electric field strength and superior thermal conductivity make SiC power device as a promising candidate for high-power and high-temperature applications, and have been extensively used in power supplies, photovoltaic converters, and smart grid [1–5]. As a typical power device, SiC PiN diode is popular due to both the large current handling capability and high blocking voltage, which is a potential choice as the freewheel diode for switch protection in high-inductance capacitor discharge systems [6, 7]. Recently, 27.5 kV SiC PiN diodes have been reported [8]. Compared to silicon diodes of same rated blocking voltage, the SiC PiN diode can remarkably reduce the on-state resistance and increase switching rate. It is well-known that carrier lifetime is a crucial parameter that governs the performance of high-voltage SiC PiN diodes [9, 10]. It will significantly determine the carrier injection efficiency under forward anode–cathode voltage, i.e., conductivity modulation effect, and thus greatly affects the on-state characteristics of SiC PiN diode [11]. The *Z*_1/2_ center from carbon vacancy in SiC lattice is considered as the main carrier lifetime killer. Therefore, the enhancement of carrier lifetime, especially in the lightly *n*-type SiC, can be successfully achieved by the carbon vacancy elimination through the injection of excess carbon atoms into the SiC drift layer, such as high temperature oxidation anneal/carbon implantation followed by carbon diffusion [12, 13]. Besides, low-energy electron irradiation can be also adopted in carrier lifetime control by point defects generation and reaction/evolution [14]. Compared to the intensive reports about the carrier lifetime modulation on the forward voltage and avalanche phenomena of SiC PiN diodes, the lifetime control on pulse current capability of SiC PiN diodes has not been reported and needs to be investigated essentially.

In this work, we present the modulation of carrier lifetime on current capability of SiC PiN diodes in a pulsed system. The carrier lifetime in drift layer of SiC PiN diodes is modulated by the production of *Z*_1/2_ center through neutron radiation with different doses. The pulse current of SiC PiN diodes with different carrier lifetime in the drift layer is evaluated quantitatively in a pulsed discharging topology. Both the static electrical performance and dynamic thermal characteristics are investigated to clarify the mechanisms for the pulse current capability of SiC PiN change as a function of carrier lifetime.

## Experimental

**Fig. 1:**
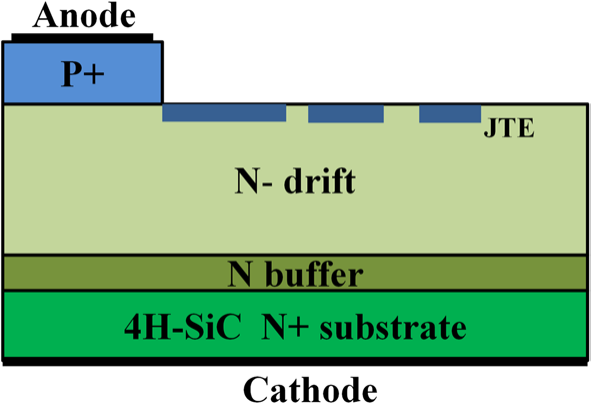
The schematic of typical SiC PiN diodes

A typical device cross-section of the fabricated SiC PiN diodes is shown in Fig. 1. The three P–i–N epitaxial layers were layer-by-layer grown on a heavily doped *n*-type 4H–SiC substrate, including a 2 μm thick *n*-buffer layer, a 90 μm thick *n*-drift layer with a light doping of 2 × 10^14^ cm^−3^, and a 2 μm top p + anode layer with a doping concentration of 2 × 10^19^ cm^−3^. The anode layer was etched using inductively coupled plasma reacting to generate isolation mesa structure. The junction termination extension (JTE) formation was then carried out using aluminum implantation to alleviate the electric field crowding near the mesa edge. The activation annealing was performed at 1650 °C in Ar ambient for 15 min. A sacrificial SiO_2_ layer was grown and then dipped with HF to remove surface defects and implantation damages. The passivation layer was formed with a 50 nm thermally grown oxide and 2 μm PECVD grown SiO_2_. Cathode and Anode were formed by Ni and Ni/Ti/Al deposition followed by rapid thermal process at 1000 oC and 800 °C, respectively. A 4 μm-thick Al overlayer was patterned on the top, while Ag film was deposited on the bottom. The thick polyimide layer was provided as a high-voltage insulation protection. Finally, the diodes were packaged in the conventional TO247 form.

4H–SiC–PiN diodes and 2 cm × 2 cm sized epitaxial wafer with i–N structure were irradiated by 1-MeV neutrons at room temperature with fluences ranging from 10^11^ to 10^13^ n/cm^2^ to modulate the carrier lifetime in 4H–SiC. Then, the carrier lifetime in the drift layer of i–N 4H–SiC epitaxial wafer as a function of neutron dose was measured at room temperature by Semilabs WT-2000 microwave photoconductive decay (μ-PCD). Neutron irradiation-induced point defects in SiC PiN diodes were characterized by a PhysTech Fourier Transform DLTS system. The applied reverse bias *V*_R_ and the filling pulse voltage *V*_P_ were − 10 V and − 1 V, respectively. The sampling period *t*_w_ of the bias pulse from *V*_R_ to *V*_P_ was 0.2 s.

**Fig. 2:**
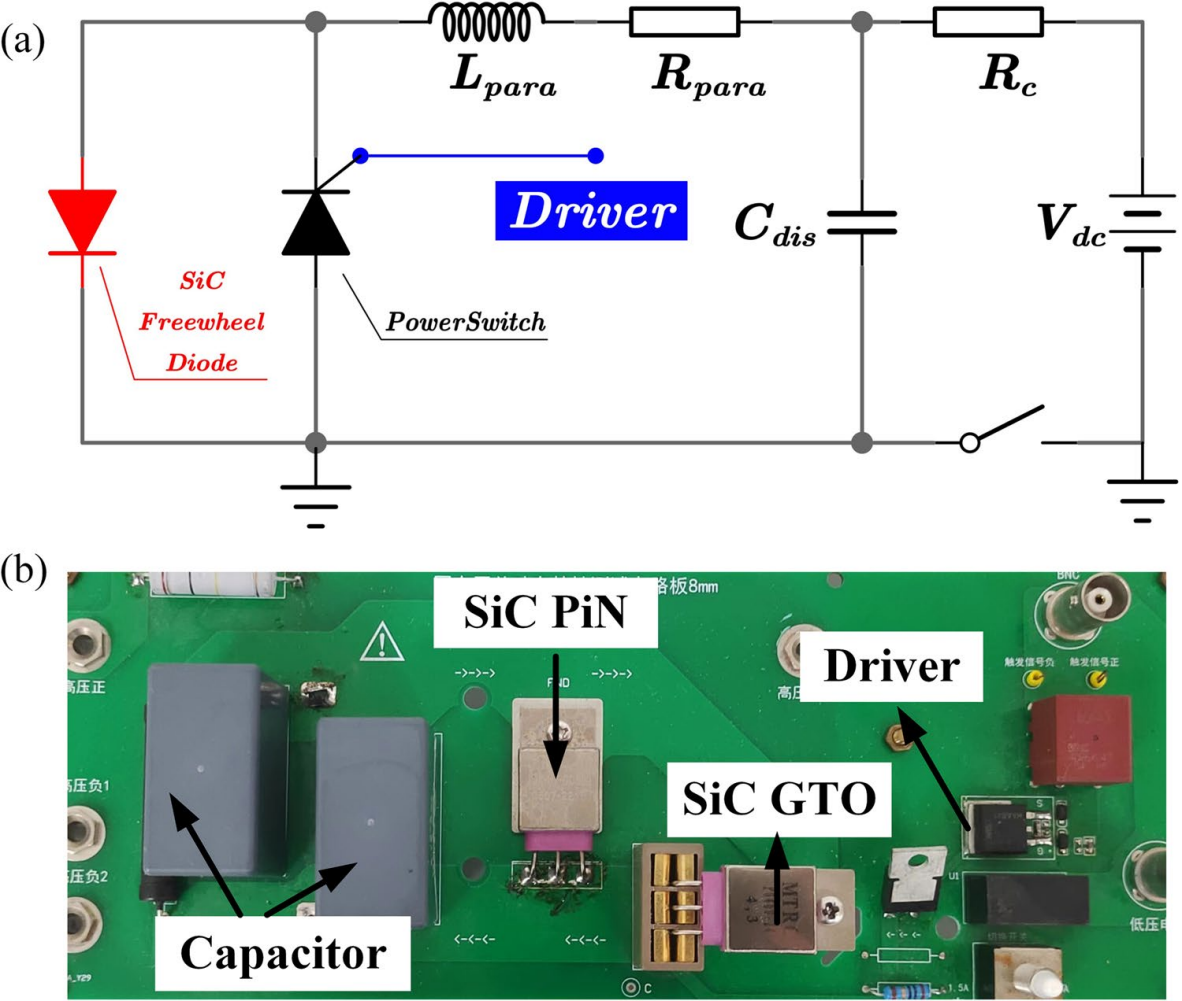
**a** Schematic circuit diagram for evaluating the pulse current capability of SiC PiN diode and **b** a photographic image of the test setup

Then, the influence of carrier lifetime on the pulse current capability of SiC PiN diodes is evaluated quantitatively with a *RLC* circuit as shown in Fig. 2a. The test circuit includes a 0.4 μF storage capacitor, the tested freewheel SiC PiN diode, SiC gate turn-off thyristor (GTO) as a power switch, and high-voltage power supply, as well as the parasitism resistor (*R*_para_) and parasitism inductance (*L*_para_). Gating SiC GTO initiates energy discharge from the capacitor, and then, the energy is dissipated into a resonant *LC* circuit. SiC PiN Diode is anti-parallel connected with the SiC GTO as the freewheeling diode to protect the asymmetric SiC GTO from the reverse voltage swing. A photograph of the test setup is shown in Fig. 2b. To clarify the influence mechanism of carrier lifetime on the pulse current capability of SiC PiN diodes, the on-state/blocking characteristics and dynamic thermal simulations by Silvaco TCAD were performed for PiN diodes with different carrier lifetime in the drift layer. The blocking and on-state characteristics of SiC PiN diodes were recorded by Keysight B1505A semiconductor characterization system at room temperature.

## Results and discussion

### Carrier lifetime modulation in neutron radiated SiC PiN diodes

**Fig. 3:**
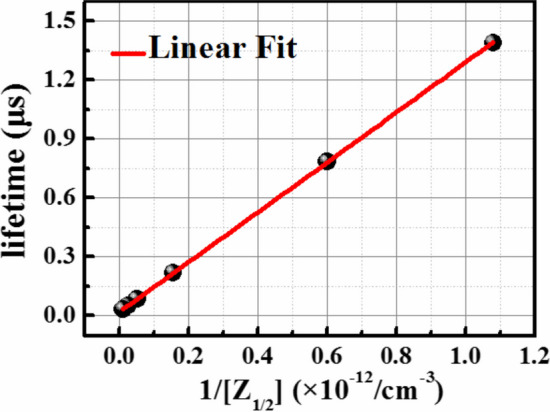
The linear dependence of carrier lifetime on the reciprocal of *Z*_1/2_ center concentration

The point defects generation in neutron irradiated SiC PiN diodes is investigated by DLTS characterization. Similar DLTS spectra are detected as Fig. 3 in ref. [15]. Two deep levels are found in the DLTS spectra in both as-grown and neutron irradiated SiC PiN diodes. From the Arrhenius plots, the derived energy level of the two traps are *E*_C_- 0.62 eV and *E*_C_- 1.55 eV, respectively, and the corresponding capture cross-sections are determined to be 7.3 × 10^–14^ cm^−2^ and 1.1 × 10^–12^ cm^−2^, respectively. These values are in good agreement with the widely reported ones of *Z*_1/2_ and EH_6/7_ center [16, 17], which both originate from carbon vacancy in the SiC lattice. Combining with DFT calculation and SRH recombination model under high-injection conditions [18], we found that the shallow *Z*_1/2_ center is dominant in carrier lifetime over the deep EH_6/7_ defects, which is resulted from the much smaller hole capture cross section of EH_6/7_ defects. Figure 3 shows the change of carrier lifetime as a function *Z*_1/2_ center concentration in the drift layer of SiC PiN diodes, which is controlled by neutron irradiation dose. It is found that the carrier lifetime shows a linear dependence on the reciprocal of *Z*_1/2_ center concentration, meaning that the carrier lifetime is governed by *Z*_1/2_ center. The carrier lifetime significantly decreases from 1.39 to 0.03 μs with the increasing *Z*_1/2_ concentration from 9.2 × 10^11^ to 9.4 × 10^13^/cm^3^, corresponding to neutron doses ranging from 10^11^ to 10^13^ n/cm^2^. In a word, the carrier lifetime can be modulated by the generation of *Z*_1/2_ center through neutron irradiation.

### Pulse current capability of SiC PiN diodes with different carrier lifetime

**Fig. 4:**
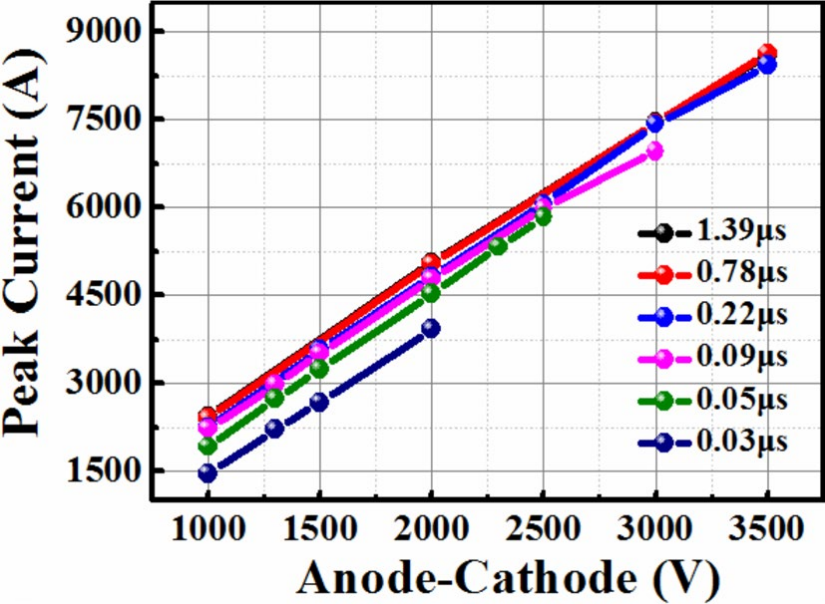
The pulse current of SiC PiN diodes in different carrier lifetime

The high pulse currents are carried out on the SiC PiN diodes in different carrier lifetime. In the anti-parallel configuration, SiC PiN diode is required to block the transient voltage on the SiC GTO to protect the switch considering its low reverse breakdown voltage, and clamps the negative sinusoidal current pulse generated by the capacitor and inductor in the circuit. Figure 4 shows the peak current *I*_peak_ as a function of carrier lifetime in PiN diodes during pulsed discharging transient tests. The pulse current is measured with the increased DC voltage until the diode get failed to turn on. Therefore the last recordable point in each curve in Fig. 4 is regarded as the maximum pulse current limit of PiN diodes with the corresponding carrier lifetime. It can be seen that the maximum *I*_peak_ is significantly promoted from 3.9 to 8.6 kA with the increasing carrier lifetime from 0.03 to 0.22 μs, while shows limited improvement when *τ* increases from 0.22 to 1.39 μs. The influence mechanism behind this phenomenon is then analysed in terms by on-state characteristics and heat generation simulation, which will be presented in the next section.

### Mechanisms for the dependence of pulse current capability on carrier lifetime

**Fig. 5:**
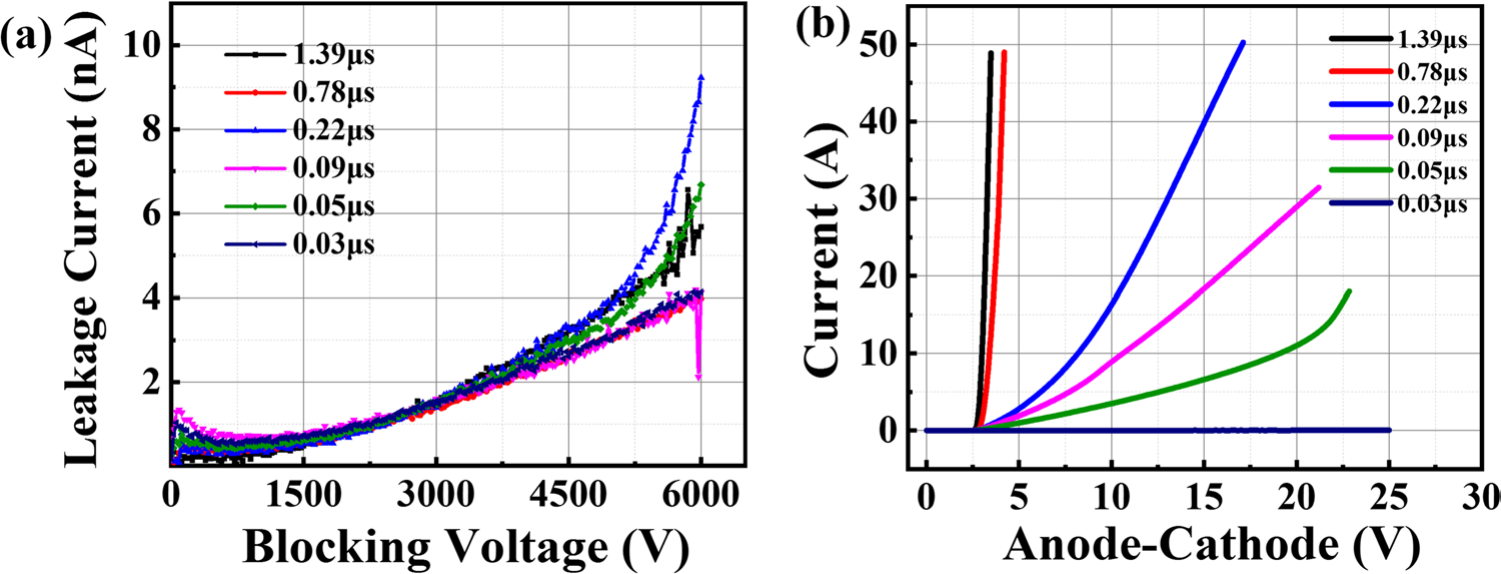
The **a** blocking characteristic and **b** on-state characteristic of SiC PiN diodes with different carrier lifetime

The blocking and on-state characteristics of SiC PiN diodes with different carrier lifetime are measured and shown in Fig. 5. The devices with different carrier lifetime are able to block 6 kV with a leakage current of less than 10nA and show no essential difference under different carrier lifetime. This can be ascribed to the wide band-gap of SiC materials, which effectively suppresses the carrier generation. Therefore, the blocking characteristics can be ruled out as the cause for the dependence of of pulse current capability on carrier lifetime. The forward voltage drop exhibits monotonous and remarkable decreases with the improved carrier lifetime as shown in Fig. 5b. At a lower carrier lifetime of 0.03 μs, the SiC PiN diode fails to turn on even with a high forward voltage of 25 V. Then, with the increase of carrier lifetime from 0.05 to 1.39 μs, the forward voltage drop at a certain current is significantly reduced, which can be ascribed to conductivity modulation effect in drift layer related with the carrier lifetime. The increased carrier lifetime suppresses the carrier recombination and thus promotes the carrier-injection efficiency into *n*-drift layer from P + anode and N + cathode sides, leading to improved conductivity modulation effect and reduced on-state resistance consequently. However, this changing trend is obviously different from the one of pulse current on carrier lifetime presented in Fig. 4.

**Fig. 6:**
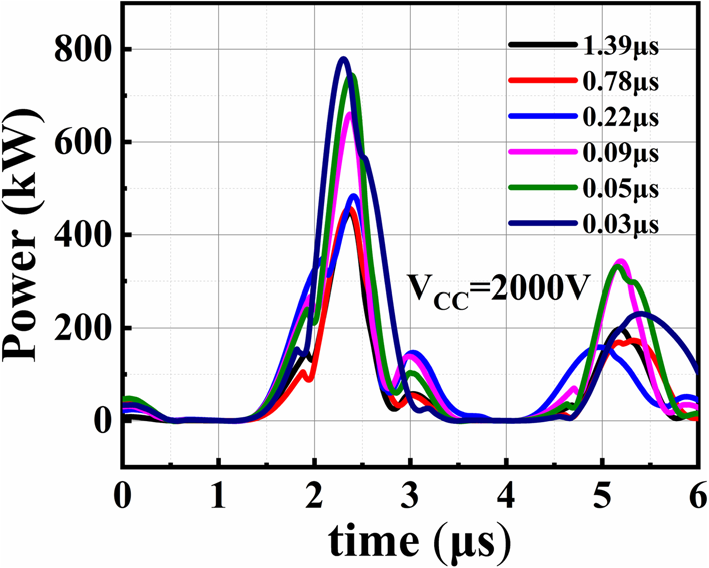
The transient power of SiC PiN diodes with different carrier lifetime

**Fig. 7:**
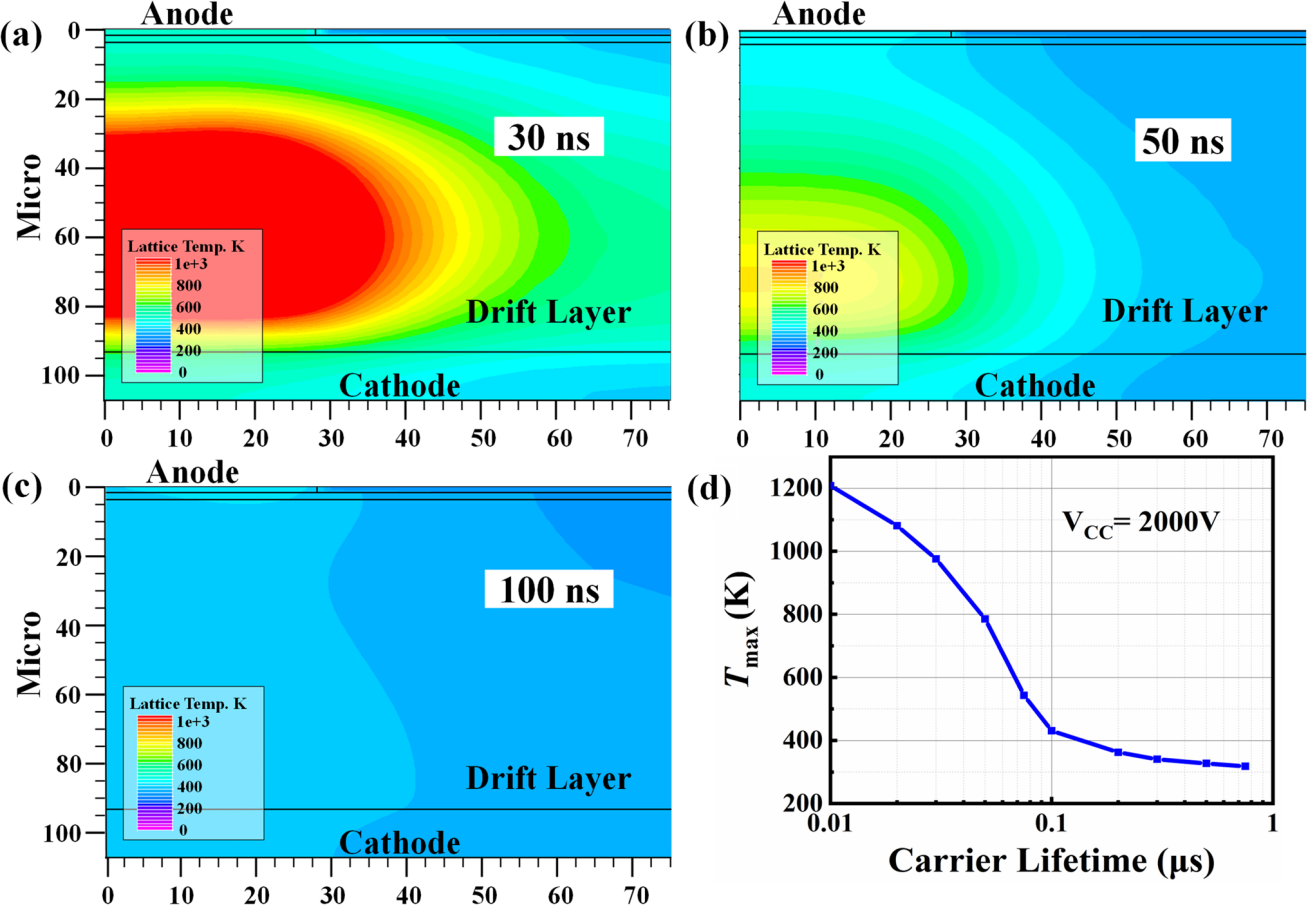
The simulated temperature distribution in SiC PiN diodes as a function of carrier lifetime in the drift layer **a** 30 ns, **b** 50 ns, **c** 100 ns and **d** *T*_max_ versus carrier lifetime

Figure 6 shows the transient power of SiC PiN diodes as a function of carrier lifetime at a pulse voltage of 2000 V. It can be seen that the transient power exhibits an insignificant increases as the decreasing carrier lifetime in the range of *τ* ≥ 0.22 μs, and is sharply increased as the carrier lifetime decreasing from 0.09 to 0.03 μs. The higher transient power at lower carrier lifetime is resulted from the significantly increased voltage drop between anode and cathode under the high pulse current, which is consistent with the deteriorative on-state characteristics in lower carrier lifetime shown in Fig. 5b. The high power in instantaneous power dissipation may generate substantial heat in the diodes and may cause damage to the metal contact or the wire bonds. Therefore, the temperature distribution in SiC PiN diodes in the pulse test is simulated as a function of carrier lifetime, as shown in Fig. 7. The maximum temperature *T*_max_ appears at the middle drift region of SiC PiN diodes, which can be ascribed to the larger resistance proportion of the drift layer. It is found that the *T*_max_ of SiC PiN diode is significantly increased with the decreasing carrier lifetime for *τ* < 50 ns, and exceeds over 1000 K for *τ* < 20 ns. Whereas the *T*_max_ is significantly reduced to lower than 400 K as carrier lifetime rises to 0.2 μs and above. Therefore, the failure reason under high pulse current tests is ascribed to temperature rise causing by the heat generation inside the device. The reduced carrier lifetime leads to the higher on-state resistance related to conductivity modulation effect, which then resulting in higher *T*_max_. However, further destructive physical analysis is needed to identify the detailed failure location using micro/nanotechnology.

## Conclusion

The dependence of pulse current capability of SiC PiN diodes on carrier lifetime has been investigated. The carrier lifetime of SiC is linearly correlated with the reciprocal of *Z*_1/2_ center concentration generated by neutron irradiation, which means that the carrier lifetime in SiC can be modulated by neutron radiation doses. With the decrease of lifetime in the range of *τ* < 0.22 μs, the pulse current capability of SiC PiN diodes is seriously deteriorated, while shows no further improvement when *τ* ≥ 0.22 μs. To clarify the influence mechanism of carrier lifetime on the pulse current capability of SiC PiN diodes, the on-state/blocking characteristics and simulation were performed for PiN diodes with different carrier lifetime in the drift layer. The dependence of pulse current capability on carrier lifetime is analysed in terms of on-state/blocking characteristics and heat generation simulation. The results reveal that the on-state resistance exhibits monotonous and remarkable decreases with the improved carrier lifetime. However, the heat generation (i.e., *T*_max_) from transient power is obviously aggravated when *τ* < 0.1 μs, while is significantly suppressed as carrier lifetime rises to 0.2 μs and above. Therefore, the carrier modulation effects on pulse current capability of SiC PiN diodes can be attributed to the heat generation resulting from the on-resistance related with conductivity modulation effect, which is accompanied with the temperature rise and hence causes the failure of SiC PiN diodes under high pulse current.

## Data Availability

The data and materials are available of this article.
